# Randomized, Open-Label Study of the Pharmacokinetics and Safety of Oral and Intravenous Administration of Omadacycline to Healthy Subjects

**DOI:** 10.1128/AAC.01393-16

**Published:** 2016-11-21

**Authors:** Haiying Sun, Lillian Ting, Surendra Machineni, Jens Praestgaard, Andreas Kuemmell, Daniel S. Stein, Gangadhar Sunkara, Steven J. Kovacs, Stephen Villano, S. Ken Tanaka

**Affiliations:** a Novartis Institute for Biomedical Research, Novartis Pharmaceuticals, East Hanover, New Jersey, USA; b Novartis Healthcare, Ltd., Hyderabad, India; c Paratek Pharmaceuticals, Boston, Massachusetts, USA

## Abstract

Omadacycline is a first-in-class aminomethylcycline antibiotic with microbiological activity against Gram-positive and Gram-negative aerobes and anaerobes and atypical bacteria that is being developed for the treatment of acute bacterial skin and skin structure infections (ABSSSI) and community-acquired bacterial pneumonia (CABP). The bioavailability of a phase 3 tablet formulation relative to that obtained via intravenous (i.v.) administration (and of other oral formulations relative to that of the phase 3 tablet) was investigated in an open-label, randomized, four-period, crossover study with healthy subjects age 18 to 50 years. Subjects received omadacycline at 100 mg i.v., 300 mg orally as two different tablet formulations with different dissolution profiles, and 300 mg as an oral solution. Plasma omadacycline concentrations were determined using a validated liquid chromatography-tandem mass spectrometry (LC-MS/MS) method. Twenty of 24 subjects completed all treatment periods. The two tablet formulations produced equivalent total exposures. The phase 3 tablet produced an exposure equivalent to that of the 100-mg i.v. dose, with a geometric mean ratio (90% confidence intervals [CI]) for area under the concentration-time curve from 0 h to infinity [AUC_∞_]) of 1.00 (0.93, 1.07). The absolute bioavailability of the tablets was approximately 34.5%. Intersubject variability was consistent among the oral formulations (∼20 to 25%). Single oral and i.v. doses of omadacycline were well tolerated; three subjects experienced mild adverse events (dizziness, nausea, and vomiting) that resolved without intervention. A 300-mg dose of the tablet formulation of omadacycline intended for use in phase 3 studies produced a total exposure equivalent to that of a 100-mg i.v. dose.

## INTRODUCTION

Omadacycline is a first-in-class aminomethylcycline antibiotic that exhibits activity *in vitro* against Gram-positive and Gram-negative aerobes and anaerobes as well as atypical bacteria (1–4). In addition, omadacycline is active against bacterial pathogens that express the two forms of tetracycline resistance, i.e., efflux and ribosomal protection (5). Based on nonclinical *in vitro* and *in vivo* investigations for antibacterial agents, the AUC/MIC ratio was determined to be the pharmacokinetic/pharmacodynamic (PK/PD) index that best predicts the efficacy of omadacycline (6). In a phase 2 study with patients with complicated skin and skin structure infections, omadacycline demonstrated efficacy and tolerability that were comparable to those of linezolid (7). Clinical development is proceeding to evaluate omadacycline as a treatment for acute bacterial skin and skin structure infections (ABSSSI) and community-acquired bacterial pneumonia (CABP). Both intravenous (i.v.) and oral formulations are available, and the proposed therapeutic dosage is 100 mg i.v. once daily or 300 mg orally once daily.

Previous PK studies with healthy subjects conducted with earlier oral formulations of omadacycline reported a time to peak concentration (*T*_max_) of 1 to 4 h, a terminal half-life (*t*_1/2_) of 17 to 18 h, a favorable AUC/MIC ratio that support once-daily dosing, and an oral bioavailability of approximately 33% (8, 9). Therefore, a 300-mg oral dose was expected to provide consistent total omadacycline exposure as measured by AUC relative to a 100-mg i.v. dose. Earlier oral capsule and table formulations of omadacycline were used in clinical studies, but a new oral tablet formulation (containing a more stable tosylate polymorph) was developed in an attempt to improve oral absorption. Another tablet formulation (here called slow-dissolution tablet) also was created using relatively high compression and a minor change in excipients, in an attempt to prolong absorption. This study evaluated the PK of these tablet formulations and of an oral solution and an i.v. infusion of omadacycline, with a focus on the bioavailability of the new oral tablet formulation intended for use in phase 3 studies.

(This research was previously presented as poster P1423 at the 21st European Congress on Clinical Microbiology and Infectious Diseases [ECCMID], 7 to 10 May 2011, Milan, Italy.)

## MATERIALS AND METHODS

This study was conducted according to good clinical practices and the ethical principles of the Declaration of Helsinki. The study was reviewed and approved by an independent institutional review board and conducted at a single center. Each participating subject provided written informed consent prior to enrollment.

### Study design.

This was an open-label, randomized, four-period, crossover study with healthy adult subjects, who were screened no more than 21 days prior to enrollment. There were four treatment periods (each separated by a washout of at least 7 days) and a study completion evaluation approximately 1 week after the last dose administration. Subjects were admitted to the clinical research center the day prior to dosing for a baseline evaluation. Eligible subjects were randomized to one of four treatment sequences, each consisting of four treatment groups: omadacycline at 100 mg via i.v. infusion over 30 min, 300 mg (2 × 150 mg) as an intended phase 3 tablet, 300 mg (2 × 150 mg) as a slow-dissolution tablet, and 300 mg as an oral solution (created using the contents of 3 × 100-mg i.v. infusion vials). On day 1 of each treatment period, subjects were given a single dose of omadacycline. Subjects remained in the clinical research center for 24 h after each dose, and pharmacokinetic assessments were conducted for up to 48 h after each dose.

### Subject selection.

Healthy men or women aged 18 to 50 years in good health, with a body mass index (BMI) within 18 to 29 kg/m^2^ and body weight at least 50 kg, were eligible. Vital signs had to be within normal ranges, with no evidence of orthostatic hypotension. Subjects were excluded for use of any investigational drug, prescription drug, over-the-counter drug, or dietary/herbal supplements within the previous 4 weeks; use of tobacco products within the previous 3 months; illicit drug or alcohol abuse with the previous 12 months; abnormally low hemoglobin; clinically significant electrocardiogram (ECG) abnormalities; or presence of any medical condition that could interfere with the conduct of the study. Pregnant or lactating women were excluded, and women of childbearing potential were required to use an approved form of contraception during the study.

### Study assessments.

Peripheral blood samples were collected predose and at 0.25 (i.v. dose only), 0.5, 1, 1.5, 2, 2.5 (oral doses only), 3, 4, 6, 8, 12, 24, and 48 h after each dose for measuring plasma omadacycline concentrations. Plasma concentrations of omadacycline were determined (Quest Diagnostics, Cambridge, MA) with the use of a validated liquid chromatography-tandem mass spectrometry (LC-MS/MS) assay having a lower limit of quantitation (LLOQ) of approximately 20 ng/ml. Calibration standard responses were linear over the range of 20 to 2,000 ng/ml. The interday assay accuracy, expressed as percent relative error for quality control (QC) concentrations, ranged from −5.3% to 2.0% in QC samples. Assay precision, expressed as the interday percent coefficients of variation (CV) of the mean estimated concentrations of QC samples, ranged from 3.1% to 4.5%.

Safety assessments consisted of noting all adverse events (AEs) and serious adverse events (SAEs), including their severity and relationship to the study drug.

At each baseline visit (the day prior to dosing) in each treatment period, physical examination, notation of weight and vital signs, 12-lead ECG, and laboratory assessments (hematology and blood chemistry) were performed. In addition, for each treatment period, blood pressure and heart rate were assessed multiple times on day 1 and again on day 2, ECG was performed twice on day 1 and again on day 2, and hematology and blood chemistry were performed on day 2. No dairy products, antacids, or other aluminum or calcium-containing products were allowed on the day of dosing.

### Statistical analysis.

All subjects who received at least one dose of study drug were included in the safety analysis. Subjects with evaluable PK data were included in the PK population. Pharmacokinetic parameters included area under the concentration-time curve from time zero to the last quantifiable concentration point (AUC_last_), area under the concentration-time curve from time zero to infinity (AUC_∞_), maximum drug concentration (*C*_max_), time to reach peak concentration following drug administration (*T*_max_), and terminal elimination half-life (*t*_1/2_). Pharmacokinetic parameters were determined from a noncompartmental method(s) using WinNonlin Pro (version 5.2). Primary comparisons were made between AUC_last_ and AUC_∞_ for the i.v. and intended phase 3 oral tablets. Absolute bioavailability (*F*) was determined by the equation *F* = (AUC_∞_ [oral] × dose [i.v.])/(AUC_∞_ [i.v.] × dose [oral]). In addition, secondary comparisons were made for AUC_last_, AUC_∞_, *C*_max_, and *T*_max_ between the oral formulations.

A linear mixed-effects model analysis was performed on log-transformed AUC and *C*_max_ of omadacycline. Sequence, period, and treatment were included as fixed effects, while subject nested within sequence was included as a random effect in the model. The point estimates and the associated 90% confidence intervals (CI) for the test/reference ratios were calculated by exponentiation of the differences of mean logarithms (test − reference) obtained from the linear mixed-effects model analysis. Data were reported as geometric mean and 90% CI for comparison of formulations for AUC_last_, AUC_∞_, and *C*_max_. Coefficient of variation was determined for all PK parameters.

## RESULTS

Twenty-four subjects were randomized, and 20 subjects completed all study periods. Four subjects were discontinued because of protocol violations during the study (two for alcohol use and two for tobacco use). Based on the treatment periods that were completed before these discontinuations, a total of 21 or 22 subjects provided PK data for each of the treatments. The age (mean ± standard deviation) of all subjects was 36.0 ± 10.9 years, the mean weight was 75.8 ± 11.5 kg, the mean BMI was 24.6 ± 3.4 kg/m^2^, and 23 (96%) subjects were male (Table 1).

**TABLE 1:** Baseline demographics of patients in this study

Parameter	Value for subjects (*n* = 24)
Age, yrs	
Mean ± SD	36.0 ± 10.9
Median	38.5
Range	18–50
Height, cm	
Mean ± SD	175.5 ± 7.3
Median	175.5
Range	161–169
Wt, kg	
Mean ± SD	75.8 ± 11.5
Median	74.0
Range	52–96
Body mass index, kg/m^2^	
Mean ± SD	24.6 ± 3.4
Median	25.5
Range	19.4–28.7
Male, no. (%)	23 (95.8)
Race, no. (%)	
Caucasian	10 (41.7)
African American	13 (54.2)
Asian	1 (4.1)

### Pharmacokinetic results.

A 300-mg dose of phase 3 tablets provided omadacycline exposure equivalent to that of the 100-mg i.v. infusion dose, as indicated by the geometric mean ratios (90% CI) for AUC_∞_ and AUC_last_ of 1.00 (0.93 to 1.07) and 0.98 (0.89 to 1.07), respectively (Table 2). The *C*_max_ for the 300-mg dose of phase 3 tablets was approximately one-third that of the 100-mg i.v. infusion. Based on AUC_∞_, the absolute bioavailability of the phase 3 tablets was estimated to be 34.5%.

**TABLE 2:** Geometric mean ratio and 90% confidence intervals for pharmacokinetic parameters (PK analysis set)

Parameter	Geometric mean ratio (90% confidence interval)		
	300-mg phase 3 tablet/100-mg i.v. infusion	300-mg slow-dissolution tablet/300-mg phase 3 tablet	300 mg in oral solution/300-mg phase 3 tablet^*a*^
AUC_last_ (μg · h/ml)	0.98 (0.89, 1.07)	0.96 (0.88, 1.06)	1.13 (1.02, 1.24)
AUC_∞_ (μg · h/ml)	1.00 (0.93, 1.07)	0.96 (0.90, 1.03)	1.19 (1.11, 1.28)
*C*_max_ (μg/ml)	0.31 (0.27, 0.35)	1.00 (0.88, 1.12)	1.11 (0.98, 1.25)

a Oral solution administered as 3 100-mg i.v. solution vials.

Compared with the phase 3 tablet, the slow-dissolution tablet provided equivalent omadacycline exposure based on *C*_max_, AUC_last_, and AUC_∞_. A 300-mg dose of oral solution provided omadacycline exposures that were higher than those for the same dose of phase 3 tablets, with geometric mean ratios (90% CI) for *C*_max_, AUC_last_, and AUC_∞_ of 1.11 (0.98 to 1.25), 1.13 (1.02 to 1.24), and 1.19 (1.11 to 1.28), respectively.

**FIG 1:**
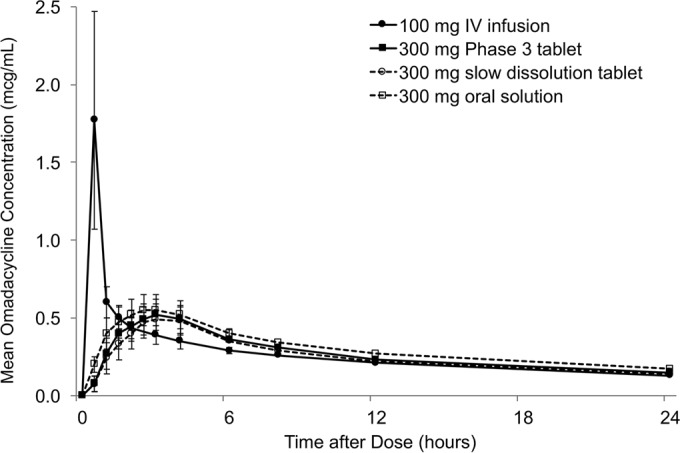
Arithmetic mean (SD) plasma concentration-time profiles of omadacycline after administration of different formulations.

The PK of the oral formulations were relatively consistent; the elimination half-life was approximately 17 h across the oral formulations (Fig. 1 and Table 3). Among the oral formulations, the *T*_max_ for omadacycline was fastest with the oral solution (median, 2.5 h), while the slow-dissolution tablet and phase 3 tablet had similar *T*_max_s (median, 3.0 h). The AUC over the 48-h sampling time (AUC_last_) accounted for over 85% of the total exposure (AUC_∞_). The intersubject variabilities in PK parameters were similar among the oral formulations: the CV was approximately 24% for AUC_∞_ and 20 to 26% for *C*_max_. For the i.v. infusion, intersubject variabilities (CV) were 16% for AUC_∞_ and 37% for *C*_max_. The mean *t*_1/2_ of 17 h was consistent between the i.v. infusion and all oral formulations. All postdose samples in all subjects in this study had quantifiable concentrations of omadacycline; only the predose plasma samples had concentrations below the assay LLOQ.

**TABLE 3:** Summary of pharmacokinetic parameters

Parameter	Omadacycline formulation and dose^*a*^			
	100 mg i.v. (*n* = 21)	300-mg phase 3 tablet (*n* = 21)	300-mg slow-dissolution tablet (*n* = 22)	300 mg in oral solution^*b*^ (*n* = 22)
AUC_last_ (μg · h/ml)	8.8 ± 1.4	8.8 ± 2.0	8.3 ± 2.0	10.0 ± 2.7
% CV	15.6	22.4	23.5	27.3
AUC_∞_ (μg · h/ml)	10.0 ± 1.5	10.3 ± 2.5	9.6 ± 2.3	11.9 ± 2.8^*c*^
% CV	15.5	24.3	23.9	23.7
*C*_max_ (μg/ml)	1.8 ± 0.7	0.5 ± 0.1	0.5 ± 0.1	0.6 ± 0.2
% CV	36.8	19.8	22.4	25.5
*T*_max_ (h)	0.5	3.0	3.0	2.5
% CV	19.8	25.8	27.7	28.2
*t*_1/2_ (h)	16.8 ± 1.6	16.8 ± 1.7	16.7 ± 1.5	16.7 ± 1.8^*c*^
% CV	9.3	10.1	8.9	11.0

a Values are means ± standard deviations, except those for *T*_max_, which are medians.

b Oral solution administered as 3 100-mg i.v. solution vials.

c *n* = 20; for 2 subjects in this group the *t*_1/2_ could not be determined and the AUC_∞_ could not be calculated.

### Safety and tolerability.

Three subjects experienced adverse events: two after receiving the oral solution (one with nausea and one with vomiting) and one after the i.v. formulation (dizziness). All events were mild, considered to be related to study medication by the investigator, and resolved the same day without intervention. No drug-related discontinuations from the study were reported, and no clinically relevant changes were observed in clinical laboratory parameters. Compared to the predose rate in each period, at any postdose time point maximum mean increases in heart rate of 12 to 15 beats/min were observed across all doses within 6 h after dose administration. Maximum changes in blood pressure were smaller in magnitude and tended to occur somewhat later, in some cases up to 12 h after dose administration; maximum mean increases in systolic pressure were 6 to 10 mm Hg and in diastolic pressure were 2 to 5 mm Hg. All changes in these vital signs were asymptomatic, and readings returned to normal levels within 24 h. There were no clinically significant ECG findings.

## DISCUSSION

A previous PK study with healthy volunteers using single omadacycline doses of 100 mg i.v. and 300 mg of an earlier oral tablet formulation (containing a different polymorph and less disintegrant than the phase 3 tablets) reported an oral bioavailability of 33% (9). Mean PK parameters for the 300-mg oral dose of that earlier tablet formulation included a *C*_max_ of 590 ng/ml, AUC_∞_ of 7.5 μg · h/ml, and *T*_max_ of 1.9 h (9). The results from the current study demonstrated that a 300-mg oral dose using the new phase 3 tablets was bioequivalent to omadacycline at 100 mg i.v. In addition, the exposures observed for the two tablets were equivalent. Therefore, although the exposure for the slow-dissolution tablet was not directly calculated relative to that of the i.v. dose, its equivalence to that of the phase 3 tablet implies that its exposure after a 300-mg oral dose was also bioequivalent to that of the 100 mg i.v. dose. The *T*_max_ for the phase 3 tablets was longer in this study than that of the earlier tablet formulation (approximately 3 h versus 1.8 h), which may reflect different absorption characteristics of the formulations or simply study-to-study variability. However, the *C*_max_ and AUC with the intended phase 3 tablets were consistent with previous reports (9). The expected dosing regimen of oral omadacycline for treatment of ABSSSI and CABP is 300 mg once daily. Since the anticipated exposure over a dosing interval at steady state will be equivalent to its AUC_∞_ following a single dose, the steady-state exposure (AUC_0–24_) for 300 mg of the phase 3 formulation administered once daily is expected to be approximately 10 μg · h/ml. Data on PK linearity across doses are limited for the phase 3 tablet, but a phase 1 study that evaluated doses of 300 mg, 450 mg, and 600 mg administered once daily for 5 days found very nearly dose-proportional AUCs across this dose range (Paratek, data on file).

The PK and intersubject variability in PK parameters of the oral formulations were consistent; intersubject variabilities in PK parameters were similar across formulations, with a CV of approximately 24% for AUC_∞_ and 20 to 26% for *C*_max_. The mean half-life of approximately 17 h across all formulations and the AUC/MIC ratio support once-daily dosing.

Omadacycline pharmacokinetics are consistent with those of some older tetracycline-related antibiotics. Both doxycycline and minocycline, which are well absorbed after oral administration, have a half-life of approximately 16 h and a *T*_max_ of about 2 h (10–12). Some newer tetracycline-derived compounds have even longer half-lives but may have lower oral absorption and bioavailability (10, 13). Tigecycline, a glycylcycline, has a half-life of approximately 22 to 66 h but demonstrates poor oral absorption and is not available as an oral formulation (10). Eravacycline is a fluorocycline antibiotic in clinical development. A phase 1 study of eravacycline in healthy volunteers found that a 300-mg oral dose was approximately comparable to a 1.5-mg/kg i.v. dose for AUC_0–24_; the half-life was 27 h. However, the absolute bioavailability was 28%, and the mean *C*_max_ was 0.3 μg/ml with the 300-mg oral dose, versus 2.8 μg/ml with the 1.5-mg/kg i.v. dose (13).

Safety analyses in this study showed that only three subjects experienced an adverse event (nausea, vomiting, or dizziness), but all such adverse events were of mild intensity and resolved the same day. Asymptomatic increases in heart rate were observed following dosing; this finding has been seen in healthy subjects in other phase 1 studies of omadacycline (9, 10, 14). In contrast to this finding in studies involving healthy subjects, no apparent effect on heart rate was observed in a phase 2 study of omadacycline for the treatment of patients with complicated skin and skin structure infections; however, the intensity of vital sign monitoring was less than used in the studies with healthy volunteers (7).

Findings from nonclinical studies suggest that omadacycline has a concentration-dependent vagolytic effect on heart rate (14). The clinical implications are consistent with potential increases in heart rate in human individuals with greater vagal tone and lower resting heart rates, such as healthy subjects participating in phase 1 clinical studies. The absence of any beta-adrenergic receptor binding and any direct inhibitory or stimulatory *ex vivo* effects of omadacycline in sinoatrial node studies suggests that if these heart increases occur in human subjects, they should not exceed the intrinsic heart rate in the absence of vagal stimulation. The small self-limited increases in blood pressure that were observed in this study were not associated with adverse events or ECG changes and are difficult to interpret in the absence of a control group. Blood pressure changes were not notable in other phase 1 studies of omadacycline.

In summary, the results from this study demonstrated that the tablet formulation containing 150 mg of omadacycline administered as a 300-mg (2 × 150 mg) dose and intended for use in phase 3 studies was bioequivalent to a 100-mg i.v. dose for the pharmacokinetic parameter (AUC) that drives its antimicrobial activity. Absolute bioavailability was 34.5% and generally consistent with *a priori* predictions that supported selection of the 300-mg oral dose. A phase 3 development program is planned using these doses to evaluate the efficacy and safety of omadacycline as a once-daily oral or intravenous therapy for treating patients with ABSSSI and CABP. The ability to switch from i.v. to oral dosing of the same drug with reliable maintenance of therapeutic exposure can be an important consideration for the clinical use of omadacycline.
